# Pericardial lipoma encasing the right coronary artery: a multimodality imaging case report

**DOI:** 10.3389/fradi.2026.1914611

**Published:** 2026-07-30

**Authors:** Xiaoyang Liu, Ziming Liu, Xiuyun Luo, Wei Miao

**Affiliations:** 1 School of Medical Imaging, Shandong Second Medical University, Weifang, China; 2 Ultrasound Department, Weifang People’s Hospital, Weifang, China

**Keywords:** case report, echocardiography, multimodality imaging, pericardial lipoma, right coronary artery

## Abstract

**Background:**

Pericardial lipomas are rare, benign cardiac tumors. Encasement of a coronary artery by such a tumor is extremely uncommon and significantly elevates surgical risk. We report the first case in which multimodality imaging clearly demonstrates a pericardial lipoma enveloping the right coronary artery (RCA) without luminal stenosis, adding a novel anatomical observation to the literature.

**Case presentation:**

A 32-year-old woman presented with a one-year history of exertional chest tightness and dyspnea that had worsened over three days. Physical examination and laboratory tests were unremarkable except for elevated thyroid-stimulating hormone (13.26 μIU/mL). Echocardiography revealed a 7.8 cm hypoechoic mass above the aortic root with a segment of the RCA coursing within it; color Doppler showed no flow disturbance. Contrast-enhanced cardiac computed tomography (CT) and cardiac magnetic resonance (CMR) confirmed a homogeneous fat-density mass (CT attenuation −85 HU, no enhancement, complete signal suppression on STIR) consistent with a pericardial lipoma. The tumor measured 5.5 cm in maximum transverse diameter and was seen to completely encase the RCA without stenosis. A diagnosis of pericardial lipoma with RCA encasement and concomitant subclinical hypothyroidism was made. After multidisciplinary discussion, levothyroxine was initiated and a staged plan including preoperative coronary angiography followed by surgical resection with RCA protection was formulated. The patient's symptoms resolved at discharge. At one- and three-month follow-ups she remained asymptomatic, but she subsequently declined all further contact and was lost to follow-up; no adverse cardiac events were recorded during the observation period.

**Conclusion:**

Pericardial lipomas can encase a coronary artery while maintaining luminal patency due to the compliance of adipose tissue. When a pericardial mass is detected on echocardiography, the coronary course must be actively traced. Multimodality imaging with CT and cardiac magnetic resonance (CMR) is essential for delineating the tumor-artery relationship and guiding surgical strategy. Coexisting endocrine disorders should be corrected preoperatively. Although the loss of long-term follow-up limits our conclusions, the imaging findings themselves provide an important cautionary lesson.

## 1 Introduction

Cardiac lipomas account for 2%–8% of benign cardiac tumors. Since most patients are asymptomatic, they are often discovered incidentally during imaging examinations, and their true incidence may be underestimated (1, 2). Pericardial lipomas typically arise from subepicardial adipose tissue and may compress cardiac structures when large. True encasement of a coronary artery, as opposed to extrinsic compression, has not been previously documented with multimodality imaging, and it fundamentally alters surgical risk and planning.

## 2 Case description

### 2.1 Timeline

A summary of the key clinical events is presented in Table 1.

**Table 1:** Timeline of key case events.

Time point	Event
1 year prior	Onset of exertional chest tightness, dyspnea, and occasional chest pain
3 days prior	Worsening of symptoms; presentation to our hospital
Hospital day 1–3	Echocardiography, contrast-enhanced cardiac CT, and cardiac MRI completed; laboratory tests revealed subclinical hypothyroidism
Hospital day 4	Endocrinology consultation; levothyroxine 12.5 μg/day initiated
Hospital day 5	Multidisciplinary discussion (cardiac surgery, cardiology, endocrinology); treatment plan formulated
Hospital day 6	Symptom relief; discharged with instructions for endocrinology follow-up and elective return for coronary angiography and surgery
1 month post-discharge	Endocrinology clinic visit; TSH decreased to 5.2 μIU/mL; no cardiovascular symptoms; levothyroxine increased to 25 μg/day
3 months post-discharge	Telephone follow-up; patient asymptomatic but had not undergone angiography or surgery; reiterated the advice
6 months post-discharge (time of writing)	All contact attempts unsuccessful; no further hospital visits; patient deemed lost to follow-up

### 2.2 Diagnostic assessment

A 32-year-old woman presented with a one-year history of exertional retrosternal chest tightness and dyspnea, which had worsened over the preceding three days. The discomfort was occasionally accompanied by chest pain that resolved with rest. She denied paroxysmal nocturnal dyspnea, orthopnea, or productive cough. Her past medical and family history was unremarkable, with no hypertension, diabetes, smoking, or alcohol use.

On examination, vital signs were within normal limits (temperature 36.2°C, pulse 80 bpm, respiratory rate 20 breaths/min, blood pressure 122/81 mmHg). Cardiac auscultation revealed a regular rhythm without murmurs or pericardial friction rub, and there were no signs of heart failure. Initial laboratory investigations, including complete blood count, coagulation profile, cardiac enzymes, and inflammatory markers, were normal. However, thyroid function tests showed an elevated thyroid-stimulating hormone level of 13.26 μIU/mL (reference range 0.35–4.94 μIU/mL) and a positive anti-thyroglobulin antibody (10.10 IU/mL), consistent with subclinical hypothyroidism.

**Figure 1:**
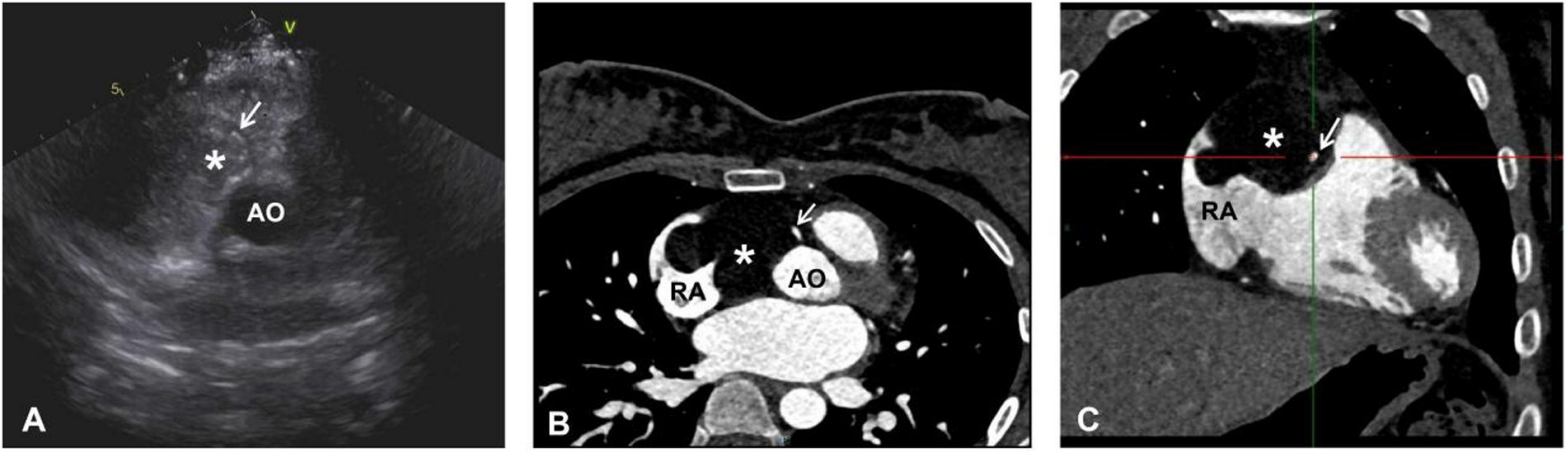
Multimodality imaging of the pericardial mass. **(A)** Transthoracic echocardiography (parasternal short-axis view) showing a hypoechoic mass (*) above the aortic root (AO), with the right coronary artery (arrow) coursing through it. **(B)** Contrast-enhanced cardiac CT (axial view) demonstrating an irregular, fat-density mass (*) within the pericardium extending toward the right atrium (RA), along with the right coronary artery (arrow) and aorta (AO). **(C)** Contrast-enhanced cardiac CT (coronal MPR reconstruction) showing the mass (*) in relation to the right atrium (RA) and right coronary artery (arrow).

Transthoracic echocardiography (GE VIVID E95 M5Sc probe, 1–5 MHz) showed a large, homogeneous, hypoechoic mass measuring 7.8 × 3.6 × 4.7 cm located above the aortic root and compressing the right atrium. A segment of the right coronary artery (RCA) was seen traversing the mass; color Doppler demonstrated normal flow without turbulence or acceleration (Figure 1A). Cardiac chamber sizes, wall motion, and valvular function were essentially normal.

Contrast-enhanced cardiac CT (Siemens SOMATOM Drive) confirmed an irregular, purely fat-density mass (CT attenuation −85 HU) within the pericardium, with no enhancement after contrast administration. The maximum transverse diameter was 5.5 cm. Multiplanar reconstruction (MPR) clearly depicted the RCA entirely enveloped by the tumor, with no luminal narrowing or evidence of invasion (Figures 1B,C).

**Figure 2:**
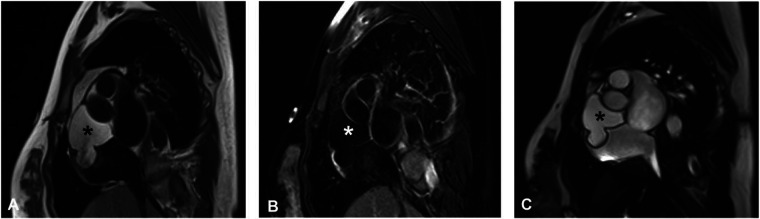
Characterization of the lipoma by CMR. **(A)** T1-weighted imaging demonstrates a well-circumscribed hyperintense mass (*) in the right pericardial region. **(B)** Fat-suppressed (STIR) sequence shows complete signal attenuation of the lesion (*). **(C)** Post-contrast T1-weighted imaging shows no enhancement of the mass (*).

Cardiac magnetic resonance imaging (Siemens MAGNETOM Prisma 3.0T) further characterized the lesion: it was hyperintense on T1-weighted images, the signal was completely suppressed on STIR sequences, and post-contrast imaging showed no enhancement-features pathognomonic for a simple lipoma. The RCA was again seen coursing through the mass with a normal caliber. Left ventricular ejection fraction was 58% (Figure 2). Detailed equipment information is provided in Table 2.

**Table 2:** Equipment list.

Equipment category	Manufacturer	Model/Specification	Purpose
Ultrasound System	GE	VIVID E95	Cardiac ultrasound examination
Ultrasound Probe	GE	M5Sc (1–5 MHz)	Transthoracic cardiac imaging
CT Scanner	Siemens	SOMATOM Drive (dual-source)	Contrast-enhanced cardiac CT
MRI Scanner	Siemens	MAGNETOM Prisma 3.0T	Cardiac magnetic resonance
Image Post-processing Workstation	Carestream Health	Carestream Vue PACS	Multiplanar reconstruction

The characteristic imaging features—homogeneous fat density, complete signal suppression on STIR, and absence of contrast enhancement—confirmed the diagnosis of a pericardial lipoma. Other entities such as pericardial effusion, mediastinal lipoma, liposarcoma, and pericardial fat pad hyperplasia were easily excluded.

### 2.3 Treatment and follow-up

An endocrinology consultation recommended initiation of levothyroxine 12.5 μg/day orally, with the goal of normalising thyroid function before any cardiac intervention, in accordance with ATA guidelines.

Following multidisciplinary discussion among cardiac surgery, cardiology, and endocrinology, a staged management plan was formulated: medical correction of hypothyroidism, preoperative coronary angiography to delineate the tumor–RCA relationship, and surgical resection with careful dissection of the RCA, with readiness for coronary artery bypass grafting if intraoperative separation proved unsafe.

The patient was advised to maintain a normal dietary iodine intake and to avoid iodine-containing supplements, as excessive iodine may exacerbate underlying thyroiditis and delay the achievement of euthyroidism. She was also advised to attend regular endocrinology follow-up for dose titration.

### 2.4 Follow-up and outcomes

At the time of discharge, her exertional chest tightness and dyspnea had markedly improved. One month later, at an endocrinology outpatient visit, her TSH had declined to 5.2 μIU/mL, and levothyroxine was increased to 25 μg/day. She reported no cardiovascular symptoms, and her physical examination and electrocardiogram were unremarkable. She was again counseled on the importance of timely angiography and surgery.

A telephone follow-up at three months post-discharge revealed that she had resumed normal activities and remained asymptomatic but had not yet undergone any of the recommended cardiac procedures. She acknowledged the advice and agreed to arrange them. However, thereafter, all attempts at contact (by telephone and WeChat) were unsuccessful, and a review of the hospital information system revealed no subsequent visits. Approximately six months after discharge, she was deemed lost to follow-up.

During the period of observation, no adverse cardiac events or medication-related side effects were reported. Adherence to levothyroxine was good based on the one-month laboratory results, but adherence to the cardiac work-up and surgical plan could not be assessed.

### 2.5 Patient perspective and informed consent

During her hospitalization and at the one-month follow-up visit, the patient expressed understanding and willingness regarding the use of her de-identified clinical data and images for academic publication. She provided written informed consent to this effect. No further patient-reported perspectives were available after loss to follow-up. The signed consent form remains on file.

## 3 Discussion

This case is, to our knowledge, the first reported instance in which multimodality imaging-echocardiography, CT, and CMR-cleanly depicts a pericardial lipoma completely encasing the RCA without producing any luminal stenosis or flow disturbance. Most previously reported cases of pericardial lipomas have described symptoms arising from cardiac chamber compression or outflow tract obstruction (3, 4).When these tumors involve the coronary arteries, the literature has typically described extrinsic compression leading to luminal narrowing and myocardial ischemia (5, 6). For example, Bogdanović et al. reported a fatal acute myocardial necrosis from tumor compression in an 80-year-old woman (5), and Younes et al. described a 49-year-old woman with non-ST-elevation myocardial infarction and angiographically normal arteries, attributed to non-obstructive extrinsic mechanisms (6). In contrast, our patient's cardiac biomarkers, electrocardiogram, and wall motion showed no ischemic changes. The exertional symptoms likely arose from right atrial compression by the large tumor, which impaired venous return and limited cardiac output reserve during exercise.

Embryologically, pericardial lipomas originate from subepicardial adipose tissue and tend to grow along vascular planes. The inherent compliance of fat allows the mass to gradually envelop the vessel rather than invade it, thereby preserving luminal patency (7). This biological property explains the absence of stenosis in our patient and represents a critical anatomical variant that all imaging specialists and surgeons should recognize.

The practical implications of this finding are profound. When the tumor only extrinsically compresses the coronary artery, simple resection relieves the obstruction. However, when the artery is encased, as in this case, blind surgical dissection could lacerate the vessel, causing acute myocardial ischemia. Hence, preoperative coronary angiography and, if indicated, myocardial perfusion imaging become indispensable. The operating team must also be prepared for CABG should safe separation prove impossible. Our multidisciplinary plan, which prioritized thyroid optimization, mandatory preoperative angiography, and a hierarchical intraoperative strategy, exemplifies a patient-safety-centered approach.

The concomitant thyroid dysfunction was an additional perioperative consideration. Levothyroxine remains the standard treatment for hypothyroidism, with dose adjustment aimed at normalizing serum TSH while avoiding overtreatment (8).

### 3.1 Limitations

The most significant limitation is the loss to follow-up, which precluded coronary angiography, surgical resection, and histopathological confirmation. The diagnosis therefore rests entirely on imaging, and the long-term oncological and coronary outcomes remain unknown. Moreover, TSH had not fully normalized, and the symptom improvement could be partly attributable to rest and lifestyle modification. Nevertheless, the unique imaging documentation of RCA encasement without stenosis stands on its own as a previously unreported anatomical observation with direct implications for clinical practice.

### 3.2 Key lessons from this case

When a pericardial mass is identified on echocardiography, the sonographer must actively trace the course of the coronary arteries, especially near the atrioventricular groove and aortic root.

Multimodality imaging with CT (particularly MPR) and CMR provides a non-invasive, accurate depiction of the tumor-vessel relationship.

Coronary artery encasement dramatically increases surgical complexity; preoperative angiography and a plan for possible CABG are mandatory.

Concurrent endocrine abnormalities should be corrected before elective cardiac surgery to minimize perioperative risk.

## 4 Conclusion

Pericardial lipomas can completely encase the right coronary artery without causing luminal stenosis-an anatomical relationship that is easily overlooked on standard imaging but has critical surgical implications. Echocardiography should be supplemented by CT and CMR to delineate the coronary course, and preoperative coronary angiography should be performed whenever encasement is suspected. Coexisting conditions such as hypothyroidism should be treated before surgery. Although this case lacks long-term follow-up, the imaging findings serve as a stark reminder that careful preoperative anatomical mapping and multidisciplinary planning are essential when managing pericardial masses.

## Data Availability

The original contributions presented in the study are included in the article/Supplementary Material, further inquiries can be directed to the corresponding author.
